# The Impact of Extracorporeal Magnetic Stimulation as Addition to Mirabegron in Overactive Bladder Treatment in Women: A Single-Centre Randomized Sham-Controlled Study

**DOI:** 10.3390/jcm13030916

**Published:** 2024-02-05

**Authors:** Uros Bele, Tamara Serdinšek, Evgenija Homšak, Igor But

**Affiliations:** 1 Medical Faculty, University of Maribor, 2000 Maribor, Slovenia; uros.bele@uniklinikum.kages.at (U.B.); evgenija.homsak@ukc-mb.si (E.H.); igor.but@ukc-mb.si (I.B.); 2 Department of Urology, Medical University of Graz, 8036 Graz, Austria; 3 Department of General Gynaecology and Gynaecologic Urology, Clinic for Gynecology and Perinatology, University Medical Centre Maribor, 2000 Maribor, Slovenia; 4 Department of Laboratory Diagnostics, University Medical Centre Maribor, 2000 Maribor, Slovenia

**Keywords:** magnetic stimulation, mirabegron, overactive bladder, patient satisfaction, urge urinary incontinence, urgency

## Abstract

**(1) Background:** The purpose of our prospective, single-blinded, randomized, sham-controlled study was to investigate the effect of the additional extracorporeal magnetic stimulation (ExMI) to pharmacological treatment in overactive bladder syndrome (OAB) in women. **(2) Methods:** We recruited 56 women with OAB, who were allocated into two study groups: the active group received mirabegron 50 mg daily and a total of 16 sessions of ExMI in 8 weeks, whereas the sham group received mirabegron 50 mg daily and sham stimulation following the same treatment protocol. Treatment success was evaluated after 4 and 8 weeks. **(3) Results:** Both groups experienced significant reduction in daytime urinary frequency, nocturia, and number of weekly incontinence episodes after 8 weeks. There were no statistically significant differences in end-point daytime urinary frequency and nocturia between groups. However, the overall average reduction rate in weekly number of incontinence episodes was 43.7% in treatment group and 24.2% in the control group. The number of urinary incontinence episodes in the treatment and control group was reduced for 3.8 ± 11.8 vs. 2.5 ± 4.3 episodes at week 4 and additional 3.3 ± 6 vs. 0.4 ± 3.2 episodes at week 8, respectively (*p* = 0.013). Moreover, IIQ-7 score showed a significantly greater score reduction and patients’ evaluated improvement of symptoms was higher in the active group. **(4) Conclusions:** The addition of ExMI to mirabegron in OAB treatment further improves the weekly incontinence episode reduction rate and also leads to grater improvement in symptoms.

## 1. Introduction

The clinical syndrome of overactive bladder (OAB) is characterized by urinary urgencies with increased urinary frequency and nocturia, with or without urge urinary incontinence in the absence of urinary tract infection or other obvious pathologies [1]. This condition shows a 12.8% prevalence among the female population, with an increasing prevalence with age [2], thus resulting in an important economic impact [3]. Because of the nature of the disease, patients often experience impaired quality of life, accompanied by social isolation, low self-esteem, frustration, anxiety and depression [3]. Due to its high prevalence and the effect on the quality of life, OAB represents an important economic burden for the society. It has been evaluated that OAB-related costs in the United States of America (USA) were as high as USD 1925 per inhabitant or USD 65.9 billion in total. According to projections, these costs would increase to USD 76.2 and USD 82.6 billion until the years 2015 and 2020, respectively [4,5]. Another more recent systematic review from 2018 estimated that the total economic burden of OAB in the USA is over USD 100 billion annually [6]. It has also been evaluated that compared to a similar patient without OAB, the healthcare costs of OAB patients were more than 2.5 times larger [7]. In the future, the ageing of the general population will probably lead to a further increase in the OAB prevalence. It is thus important to increase our understanding of pathophysiology of this syndrome and to develop clinically and economically efficient treatment options.

Contemporary OAB treatment typically starts with less invasive behavioral and educational interventions, which are followed by pharmacological treatment. Typically, pharmacological treatment for OAB starts with antimuscarinics. While antimuscarinic treatment is more effective than a placebo, its ability to reduce symptoms is relatively limited [8]. Antimuscarinics often come with side effects such as dry mouth, constipation, blurred vision, fatigue, and potential cognitive decline. As a result, patient adherence is quite poor, with only 6–12% of patients still continuing the prescribed therapy after two years [9]. Various reasons contribute to this poor adherence, but most commonly, patients discontinue treatment due to inadequate therapeutic effects (41.3%) or side effects (22.4%) [10]. Another pharmacological treatment choice is beta-3 agonists, which achieve bladder relaxation by activating beta-3 receptors in the bladder wall. Mirabegron, a beta-3 receptor agonist, has demonstrated the ability to reduce the frequency of urination and urgency episodes without a significant difference in side effects compared to a placebo [11]. However, despite its fewer side effects, adherence issues with mirabegron are still comparable to those associated with antimuscarinics [12]. Therapy-resilient cases require surgical interventions, such as bladder wall injection of botulinum toxin A, sacral nerve stimulation, and rarely, bladder augmentation or urinary diversion [13].

Because of the relatively poor efficacy of first- and second line treatments, some alternative treatment options have been investigated in the past years. One of them is extracorporeal magnetic innervation/stimulation (ExMI) therapy, which represents an alternative to pharmacological or surgical treatment of urinary incontinence [14]. It is a non-invasive, non-surgical treatment that stimulates pelvic floor muscles through the induction of an electric current with the aid of a magnetic field [15,16]. Most studies focus on its effect on treating stress urinary incontinence [16], but some authors have shown that it is as effective in treating urgency incontinence in female patients with OAB [5,17]. Furthermore, with the adjacent magnetic stimulation of the sacral roots, symptoms of urinary frequency as well as urge incontinence can improve [18].

The aim of our study was to investigate whether the addition of ExMI to pharmacological treatment with mirabegron additionally improves OAB treatment success and patient satisfaction.

## 2. Materials and Methods

We designed a single-centre, prospective, single-blinded, randomized, sham-controlled study, which was conducted at Department of General Gynaecology and Gynecological Urology, Clinic for Gynecology and Perinatology, University Medical Centre Maribor, Slovenia, between years 2019 and 2023, including a 2-year COVID-19 pandemic gap.

This study followed the principles embodied in the Declaration of Helsinki. Research protocol was developed following the CONSORT guidelines [19]. Prior to patients’ enrolment, the trial was registered as an internal research project at our institution (project number IRP-2018/01-16). Study was approved by the National Medical Ethics Committee (approval number 0120-234/2018/4). The trial was also retrospectively registered on clinicaltrials.gov (registration number NCT06123364).

**Figure 1:**
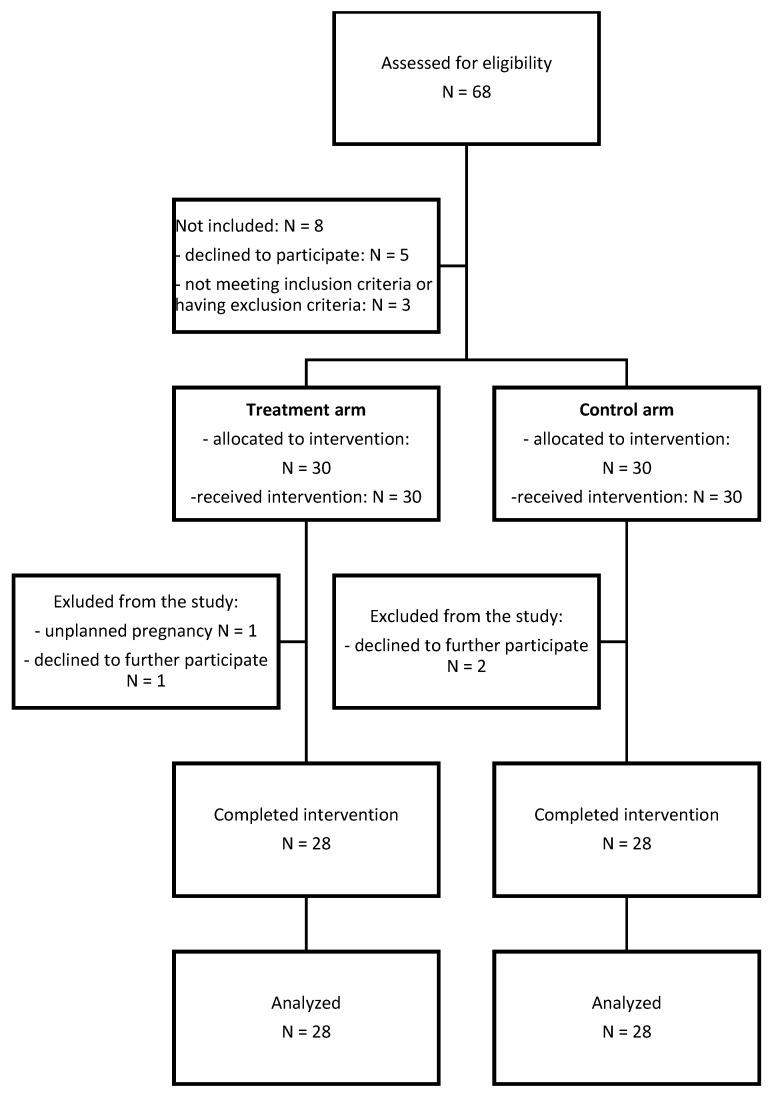
Flow chart of patient enrolment and the follow-up process.

The primary aim of this study was to evaluate whether the addition of ExMI to mirabegron (treatment arm) reduces the OAB symptoms (urgency urinary incontinence episodes, daytime frequency, nocturia), Patient Perception of Intensity of Urgency Scale (PPIUS) score, and urinary flowmetry results in comparison to treatment with mirabegron and sham ExMI (control arm). Secondary aim of the study was to investigate the effect of the addition of ExMI to mirabegron on patients’ quality of life, Incontinence Impact Questionnaire—short form (IIQ-7) score, and Urogenital Distress Inventory—short form (UDI-6) score compared to the control arm. Figure 1 shows a flow chart of the patient enrolment and the follow-up process.

We included women with OAB who fulfilled the following inclusion criteria: (i) aged between 30 and 80 years, (ii) experiencing OAB symptoms, and (iii) agreed to participate in the study. The diagnosis of OAB was made based on the patient’s visit at our urogynecology clinic, where initial diagnostics were performed. Exclusion criteria included positive urine culture or urinary tract infection, treatment with anticholinergics or mirabegron in the last 3 months, contraindications for treatment with mirabegron, pelvic floor muscles therapy (e.g., pelvic floor exercises, electrical stimulation, etc.) in the last 3 months, stress urinary incontinence, pelvic malignancies, pregnancy, cardiac pacemaker or implantable cardiac defibrillator, and electronic device or metallic implant applied to areas between the lumbar region and lower extremities. Patients who discontinued the prescribed treatment during the study or initiated other medications for OAB were also excluded from the study. All patients were informed about the study protocol and signed an informed consent form.

An a priori power analysis was carried out using G*Power software v3.1 and using reference values of weekly incontinence episodes after treatment estimated by the study conducted by Yamanishi et al. [17]. With an estimated effect size d of 0.795 and by using Wilcoxon–Mann–Whitney test, the a priori sample size was calculated as 28 individuals per group in order to retain 80% statistical power at alpha set as 0.05. Considering an up to 20% drop out rate, we decided to include a total of 68 patients.

After recruitment, patients were evenly allocated to two study groups in a randomized, single-blinded, consecutive manner using computer-generated numbers. In the active group, participants received a daily dose of mirabegron 50 mg and underwent ExMI using an electromagnetic chair (Iskra Medical Magneto STYM^®^; Iskra Medical d.o.o., Ljubljana, Slovenia). The magnetic stimulation was administered twice a week for 8 consecutive weeks, following the manufacturer’s recommendations: a magnetic stimulation frequency of 10 Hz for a total of 12 s per cycle (active time 6 s, pause time 6 s), with a total therapy time of 20 min per session. In the control group, patients received a daily dose of mirabegron 50 mg and underwent a sham stimulation on the same electromagnetic chair, following the same protocol.

Before treatment, each patient underwent a detailed assessment. Urinary culture was performed to exclude urinary tract infection. They evaluated their daytime frequency, nocturia, and urinary incontinence episodes. They also completed validated questionnaires, including PPIUS, I-QOL, IIQ-7, and UDI-6. Uroflowmetry tests and post-void residual (PVR) volume measurements were performed in a standardized manner, involving the emptying of the bladder using a urinary catheter before measurement and then filling it with 250 mL of room-temperature physiological saline through the urinary catheter. Measurements were performed by a consultant urologist. Prior to these measurements, patients kept a voiding diary for three consecutive days. At weeks 4 and 8, the whole assessment was repeated, and patients also completed a patient satisfaction survey (PSS) related to the intervention.

We performed the statistical analysis using IBM^®^ SPSS^®^ Statistics, version 22. Basic patients’ characteristics were calculated using simple statistics. Non-parametric Wilcoxon Signed Ranks test and Mann–Whitney tests were used to compare numerical differences within and between groups, respectively. Chi-square/Fisher’s exact test was used to compare categorical data between groups. Statistical significance was set at *p* < 0.05.

## 3. Results

In the enrollment period, 68 women were assessed for eligibility, and 60 were included in the study (88.2% response rate). Of these, 28 women in each group completed the 8-week treatment course (either ExMI or sham) with simultaneous pharmacological treatment with mirabegron (Figure 1).

The treatment and control group were balanced with respect to most baseline characteristics: age (60.3 ± 11.9 years (range 35–78) vs. 54.9 ± 11.7 years (30–78), respectively, *p*-value = 0.101), PPIUS score (3.8 ± 0.9 vs. 3.5 ± 0.7, *p*-value = 0.097), daytime urination frequency (8.7 (range 6–13) vs. 9.0 (range 5–15), *p*-value = 0.554), nocturia (1.1 (range 0–3) vs. 1.4 (0–4), *p*-value = 0.408), maximal urinary flow (Q-max) (24.3 ± 12.5 mL/s vs. 27.9 ± 13.7, *p*-value = 0.372), and PVR (9.3 ± 26.8 mL (range 0–120) vs. 2.5 ± 7.5 (range 0–30), *p*-value = 0.293). However, patients in the treatment group experienced a statistically significantly longer duration of symptoms (mean value of 9.2 years (range 1–30) vs. 5.6 years (range 1–30), respectively, *p*-value = 0.005). Of the 28 women in each group, 26/28 (92.3%) had urge urinary incontinence (UUI). In women with UUI, mean number of weekly incontinence episodes in treatment group was 13.3 ± 16.1 and 6.7 ± 9.1 in the control group. This difference was also statistically significant (*p*-value = 0.044).

During the treatment period, there was a significant improvement in weekly incontinence episodes, daily urinary frequency, nocturia, and PPIUS score in both groups, but without impact on the urinary flowmetry results or PVR (Table 1). There were no statistically significant differences in daytime urinary frequency, nocturia and PPIUS scores between groups at the end of the treatment period.

**Figure 2:**
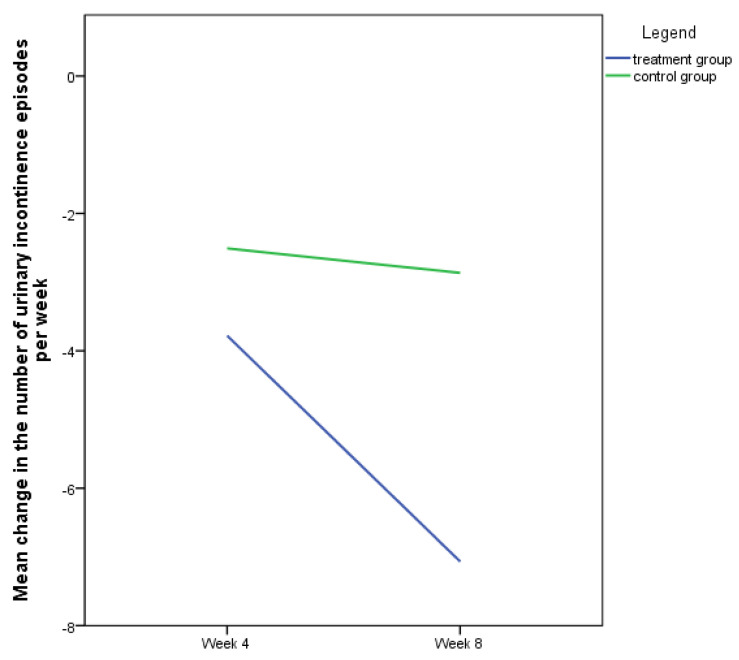
Decrease in number of weekly urinary incontinence episodes between the baseline and the follow-up visits.

However, baseline differences in number of weekly incontinence episodes precluded comparison of this variable at the end of the treatment period, which is why we decided to compare the change in the weekly number of urinary incontinence episodes between the baseline and the corresponding follow-up between both groups. As seen from Figure 2, the number of urinary incontinence episodes in the treatment group was reduced for averagely 3.8 ± 11.8 episodes at week 4 and for an additional 3.3 ± 6 episodes at week 8. In the control group, these values were 2.5 ± 4.3 and 0.4 ± 3.2 episodes, respectively. While there were no statistically significant differences in the reduction in weekly incontinence episode at week 4, this difference was statistically significant at week 8 in favor of the treatment group (*p* = 0.013). The overall average reduction rate in weekly number of incontinence episodes was 43.7% in the treatment group and 24.2% in the control group.

**Figure 3:**
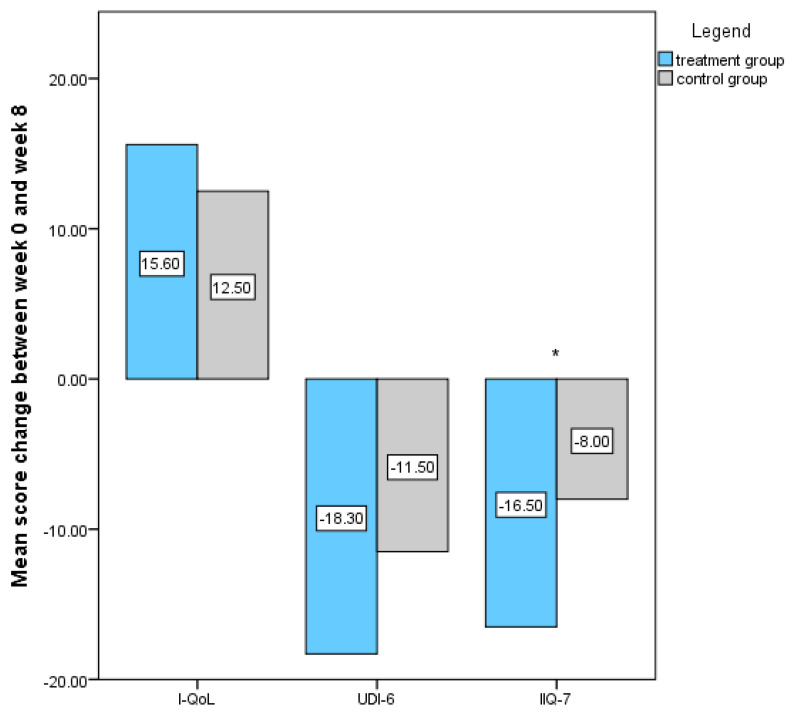
I-QoL, UDI-6, and IIQ-7 score changes between week 0 and week 8 of treatment among both study groups. Legend: *—statistically significant difference.

Scores for each individual questionnaire at the enrolment and at both follow-up visits were calculated. Table 1 shows significant improvement in all questionnaires’ scores in both groups. The mean change in questionnaire scores between week 0 and 8 between groups was statistically significant for IIQ-7 score (−16.5 ± 17.4 for treatment group and −8.0 ± 17.6, *p*-value < 0.05), whereas other values did not reach statistical significance (Figure 3). Regarding the PSS related to the intervention, 21/28 (75%) patients in the treatment group reported some improvement, and 7/28 (25%) reported substantial improvement after treatment. In the control group, these results were 14/28 (50%) and 2/28 (7.1%). The difference between groups was statistically significant (*p* < 0.001). Patients’ satisfaction rate was comparable between groups, and all patients would recommend this treatment to their friend. No serious side effects were reported in the study. However, milder side effects of treatment were reported in three patients, namely one in the treatment group and two in the control group. In the treatment group, one patient reported an unpleasant, hot skin sensation during ExMI therapy. In the control group, one patient reported an episode of cystitis, and another patient reported occasional headaches occurring after initiation of therapy. None of those patients considered the side effects so discomforting that they would want to discontinue their participation in the study.

## 4. Discussion

Magnetic stimulation has been utilized in utilized in urogynecology and other fields of medicine for quite some time. It has the advantages of being non-invasive, safe, and simple; it can directly treat the site of injury, pain and/or dysfunction; and has the ability of nerve stimulation without eliciting pain, which can be a bothersome side effect of electrical stimulation [20,21]. To the best of our knowledge, our study was the first that aimed to investigate whether the addition of ExMI to pharmacological treatment with mirabegron additionally improves treatment success in women with of OAB.

Several different studies have evaluated the effect of ExMI in OAB treatment. While some studies show that ExMI leads to short- and medium-term improvement of OAB symptoms in women and increases maximal cystometric bladder capacity [17,22,23,24], others could not confirm its beneficial effect [25,26]. A randomized placebo controlled trial from 2002 has established that ExMI of sacral roots with the aim to address the symptoms of urinary frequency and urge incontinence can result in significant symptomatic improvement following just one treatment session [18]. Similarly, one prospective trial and one retrospective study that evaluated the effects of ExMI on OAB symptoms in women observed substantial symptom improvement after ExMI treatment [27,28]. Up to this date, two systematic reviews have investigated the effect of ExMI therapy for OAB and urinary incontinence in women. A recent systematic review, published in 2023, evaluated ExMI for the treatment of female urge urinary incontinence (UUI). While the authors emphasized that the literature in this area is lacking, they also concluded that ExMI is an effective conservative method of UUI treatment [29]. Another systematic review from 2019 also indicated that ExMI is effective in urinary incontinence treatment. According to their results, ExMI reduces the frequency of urinary incontinence and improves quality of life of women with urinary incontinence [30].

Our study is the first that evaluated the addition of ExMI to pharmacological treatment with mirabegron. Moreover, searching the PubMed database, no similar study has also been performed using antimuscarinics. While both groups of our patients experienced symptom improvement, patients in the treatment group had a statistically significantly larger reduction in the number of weekly urinary incontinence episodes between weeks 4 and 8. While the reduction in the number of weekly urinary incontinence episodes in the treatment group continued even after week 4, there was a minimal additional reduction in the control group, which could suggest a cumulative effect of mirabegron and ExMI. Considering the fact that at the individual level, urgency incontinence is one of the most bothersome lower urinary tract symptoms for both men and women [31], and given the well-known psychological effects of urinary incontinence [32], our results suggest that by further reducing the number of incontinence episodes, the addition of ExMI to pharmacological treatment could further improve patients’ well-being and quality of life. A similar possibility of an additive effect has already been speculated regarding the use of ExMI in addition or after cholinergic treatment by some authors [17]. Moreover, patients’ evaluated improvement of symptoms was higher in the treatment group. Our results also show that the addition of ExMI to medical therapy with mirabegron improves some aspects of incontinence’s impact on daily activities (IIQ-7). However, based on calculated scores from the validated quality of life questionnaires used in our study, these findings do not necessarily reflect in a better quality of life.

One of the main advantages of ExMI compared to other non-pharmacological and non-surgical treatments are that it is non-invasive, atraumatic to the surrounding tissues, it has minimal side effects, and that is well-accepted by the patients [22]. Moreover, patients do not need to undress, and there is no need for vaginal or anal probes [14]. The results of our study confirm this, as all of our patients would recommend ExMI treatment to a friend with similar symptoms, regardless of whether they were treated with the active or sham ExMI.

The main advantages of our study are that it was a randomized sham-controlled study and that the sample size was calculated based on the number of urinary incontinence episodes, which was one of our primary outcomes. However, due to significant differences in number of urinary incontinence episodes between groups at inclusion despite the randomization process, we decided to compare the reduction in number of urinary incontinence episodes between groups. One of the limitations of our study is the lack of a longer follow up. Studies show that more than half of patients treated with ExMI only show recurrence of symptoms after 6 months [14]. Since our patients were treated with a combinational therapy, we do not know if this could lead to a better adherence to the pharmacological treatment and consequently to better outcomes. Another limitation is the statistically significant difference in the duration of OAB symptoms. Since patients were allocated into two study groups in a randomized, single-blind, consecutive manner, we could not influence or avert this from happening. Controversially, the treatment group, with the longer history of OAB symptoms, showed better symptom improvement in comparison to the control group with shorter duration of symptoms. This could potentially be explained with the fact that they were treated with a dual therapy (mirabegron + ExMI), or alternatively, since they have suffered from OAB symptoms longer, their expectations of treatment results may be lower. As already stated before [17], it is difficult to completely blind the patient while using an active ExMI device and the sham device, since it causes muscle contractions during activation. In our study, most patients did not have any previous experience with ExMI due to its unavailability in our public healthcare system. Patients from different study groups had their sessions on different days, so that they could not meet and exchange information. Furthermore, both devices produced the same noises and showed an identical therapy progress on the display in both study groups.

## 5. Conclusions

The results of our study show that the addition of ExMI to mirabegron in women with OAB further improves the outcome of treatment by reducing the number of urinary incontinence episodes. It also leads to greater improvement in IIQ-7 scores and higher patients’ evaluated improvement of OAB symptoms.

## Figures and Tables

**Table 1:** Comparison of study endpoints between both groups at the inclusion (week 0) and at both follow-ups (weeks 4 and 8).

	Week 0		Week 4		Week 8		Comparison within Groups (Week 0–Week 8)	
	Treatment Group *N* = 28	Control Group *N* = 28	Treatment Group *N* = 28	Control Group *N* = 28	Treatment Group *N* = 28	Control Group *N* = 28	Treatment Group *N* =28	Control Group *N* = 28
**PPIUS score (mean ± SD)**	3.8 ± 0.9	3.5 ± 0.7	3.4 ± 0.6	3.1 ± 0.9	2.9 ± 0.9	2.9 ± 0.8	<0.001 *	0.001 *
**Weekly incontinence episodes ^×^ (mean ± SD)**	13.3 ± 16.1	6.7 ± 9.1	9.5 ± 11.3	4.1 ± 7.7	6.2 ± 8.9	3.8 ± 8.1	<0.001 *	0.008 *
**Daily urinary frequency (mean, range)**	8.7 (6–13)	9.0 (5–15)	8.2 (5–13)	8.2 (5–15)	7.7 (5–13)	7.6 (5–14)	0.023 *	0.001 *
**Nocturia (mean, range)**	1.1 (0–3)	1.4 (0–4)	1 (0–3)	0.9 (0–3)	0.7 (0–3)	0.9 (0–3)	0.008 *	0.002 *
**Qmax [mL/s] (mean ± SD)**	24.3 ± 12.5	27.9 ± 13.7	23.7 ± 12.9	28.6 ± 11.4	22.9 ± 10.5	27.9 ± 10.2	NS	NS
**PVR [mL] (mean, range)**	9.3 (0–120)	2.5 (0–30)	13.7 (0–170)	0.4 ± 1.9 (0–10)	11.3 (0–110)	0 (0)	NS	NS
**I-QOL: total**	52.8 ± 27.2	58.6 ± 25.2	58.0 ± 25.8	63.0 ± 26.5	68.4 ± 23.4	71.1 ± 24,3	<0.01 *	<0.01 *
**- ALB**	50.9 ± 6.7	54.5 ± 23.6	56.4 ± 24.4	59.4 ± 24.5	67.7 ± 22.6	68.0 ± 24.0	<0.01 *	<0.01 *
**- PSI**	58.4 ± 29.1	66.1 ± 27.5	62.5 ± 28.5	69.8 ± 27.3	72.9 ± 25.9	76.6 ± 24.2	<0.01 *	<0.01 *
**- SE**	45.5 ± 29.8	52.0 ± 27.8	52.3 ± 27.3	56.6 ± 30.2	61.3 ± 24.4	66.3 ± 28.0	<0.01 *	<0.01 *
**UDI-6**	53.8 ± 21.3	44.6 ± 24.1	47.2 ± 20.3	37.5 ± 26.5	35.5 ± 21.1	33.1 ± 20.2	<0.01 *	<0.01 *
**IIQ-7**	38.9 ± 33.3	26.7 ± 30.3	28.6 ± 29.7	19.9 ± 21.6	22.4 ± 29.9	18.7 ± 26.4	<0.01 *	<0.05 *

Legend: PPIUS—patient perception of intensity of urgency scale, SD—Standard Deviation, Qmax—maximum urinary flow (mL/s), PVR—post-void residual urine volume (mL), I-QOL—Incontinence Quality of Life Questionnaire: ALB—avoidance and limiting behavior, PSI—psychosocial impacts, SE—social embarrassment; IIQ-7—Incontinence Impact Questionnaire—short form, SD—Standard Deviation, UDI-6—Urogenital Distress Inventory—short form, *—statistically significant difference, NS—no statistically significant difference, ^×^ *N* = 26 for both groups.

## Data Availability

Available on request and with regulations.
